# Harnessing Artificial Intelligence in Psychiatry: Innovations, Applications, and Ethical Considerations

**DOI:** 10.1192/j.eurpsy.2025.595

**Published:** 2025-08-26

**Authors:** A. H. I. Abu Shehab, A. B. Ciubară, S. L. Burlea, V. Doina Carina, M. Grigoraș, C.-F. Buicu, A. Ciubară

**Affiliations:** 1 Psychiatry, “Elisabeta Doamna” Psychiatry Hospital of Galati; 2 orthopaedics and traumatology, Faculty of Medicine and Pharmacy, “Dunărea de Jos” University, Galati; 3 Oral and maxillofacial surgery, 7University of Medicine and Pharmacy “Grigore T. Popa”, Iași; 4 Rheumatology; 5 Psychology, Faculty of Medicine and Pharmacy, “Dunărea de Jos” University, Galati; 6 Public health, “George Emil Palade” University of Medicine, Pharmacy, Science, and Technology, Targu Mures; 7 Psychiatry, Faculty of Medicine and Pharmacy, “Dunărea de Jos” University, Galati, Romania

## Abstract

**Introduction:**

The field of psychiatry is going through a rapid transformation as a result of the rapid advancements in artificial intelligence (AI), which are providing novel opportunities for the diagnosis, treatment, and management of mental health disorders. AI can deliver more precise and personalized mental health care by utilizing machine learning algorithms, natural language processing (NLP), and neuroimaging analysis. This paper investigates the current applications of AI in psychiatry, its prospective benefits, and the challenges that must be overcome in order to effectively integrate AI into clinical practice.

**Objectives:**

To investigate contemporary advancements in artificial intelligence (AI) applications in psychiatry, emphasizing enhancements in diagnostic precision, therapeutic personalization, and ethical considerations for integration into clinical practice.

**Methods:**

A thorough examination of AI applications in psychiatry was performed, encompassing AI utilization for psychiatric diagnosis, digital mental health therapies, neuroimaging analysis, and suicide risk assessment.

**Results:**

Artificial intelligence demonstrated considerable potential in enhancing diagnostic precision, especially via technologies such as natural language processing for evaluating speech and text, and machine learning algorithms for assessing brain imaging. AI-driven chatbots and virtual therapists shown effectiveness in administering cognitive behavioral therapy (CBT) and facilitating continuous mental health care. Predictive algorithms for suicide risk and digital phenotyping instruments present opportunities for early intervention. Nonetheless, obstacles including algorithmic bias, data security issues, and the necessity for human oversight were identified as barriers to full implementation.

**Conclusions:**

Artificial intelligence possesses revolutionary potential in psychiatry, facilitating earlier diagnoses, more individualized treatment strategies, and continuous monitoring of mental health disorders. For AI to be included into standard psychiatric care, continuous efforts are required to guarantee ethical implementation, mitigate algorithmic bias, and preserve the vital human connection in psychiatric therapy. Collaboration among AI engineers, physicians, and ethicists will be essential to fully leverage AI’s potential while ensuring the safety of patients.

**Disclosure of Interest:**

None Declared

